# Sooty bark disease of maples: the risk for hypersensitivity pneumonitis by fungal spores not only for woodman

**DOI:** 10.1186/s12995-021-00292-5

**Published:** 2021-01-21

**Authors:** Markus Braun, Doris Klingelhöfer, David A. Groneberg

**Affiliations:** Institute of Occupational Medicine, Social Medicine and Environmental Medicine, Goethe University Frankfurt, Theodor-Stern-Kai 7, D-60590 Frankfurt am Main, Germany

**Keywords:** Maple bark disease, Maple bark strippers’ lung, Cryptostroma corticale, Coniosporium corticale, Sycamore, *Acer platanoides*, Hyper allergenic spores, Extrinsic allergic alveolitis, EAA, Climate change

## Abstract

In the middle of the twentieth century, the from North America sooty bark disease (SBD) of maples was first discovered in England and has spread in the last decades in Central Europe, in particular. The trigger of SBD is the mould fungus Cryptostroma (C.) corticale. The most common infested maple is the sycamore, *Acer pseudoplatanus*, a common tree in woods and parks. The disease is characterised by peeling of the outer layer of the bark and brownish-black spores under the peeled off bark. These spores can cause maple bark disease (MBD) in humans, a hypersensitivity pneumonitis (HP) with similar symptoms like COPD, allergic asthma, influenza or flu-like infections and interstitial pneumonia. Persons who have intensive respectively occupational contact with infested trees or wood, e.g., woodman, foresters, sawyers or paper mill workers, are at risk in particular. Since C. corticale favours hot summers and host trees weakened by drought, SBD will increasingly spread in the future due to ongoing climate change. Consequently, the risk of developing MBD will increase, too. As with all HPs, e.g., farmer’s lung and pigeon breeder’s disease, the diagnosis of MBD is intricate because it has no clear distinguishing characteristics compared to other interstitial lung diseases. Therefore, the establishment of consistent diagnosis guidelines is required. For correct diagnosis and successful therapy, multidisciplinary expertise including pulmonologists, radiologists, pathologists and occupational physicians is recommended. If MBD is diagnosed in time, the removal of the triggering fungus or the infested maple wood leads to complete recovery in most cases. Chronic HP can lead to lung fibrosis and a total loss of lung function culminating in death. HP and, thus, MBD, is a disease with a very high occupational amount. To avoid contact with spores of C. corticale, persons working on infested wood or trees have to wear personal protective equipment. To protect the public, areas with infested maples have to be cordoned off, and the trees should be removed. This is also for impeding further spreading of the spores.

## Introduction

The sooty bark disease (SBD) originated in North America is a lethal fungal disease of maples, particularly of the sycamore, *Acer pseudoplatanus*. Less common, C. corticale can also infest the maple species *A. platanoides*, *A. campestre* and *A. negundo* [1]. *A. saccharum* is another common host in North America [2]. In Europe, it was first discovered on a sycamore in Wanstead Park in Essex, England, in 1945. The most apparent symptoms on affected trees are shedding of the bark and the underlying brownish-black spores of the fungus Cryptostroma (C.) corticale [3, 4].

Already in 1932, the first report in the literature on the effect of C. corticale on humans described five cases of diffuse lung disease in lumber workers peeling mould-infested maple trunks in Michigan, USA [5]. The authors reported the symptoms as typically asthmatic and identified the fungus as Coniosporium corticale, later named Cryptostroma corticale [4]. In 1962, a case report was published in the New England Journal of Medicine reporting the maple bark disease (MBD) as a pneumonitis caused by C. corticale [6].

After the first European record of C. corticale in 1945 in Essex, England, the fungus was occasionally found in the more southern parts of England (e.g. Greater London, Somerset, Norfolk and Devon) [7]. In 1950, but also in 1991–1992 and 2005 after dry periods, C. corticale was detected in the surroundings of Paris, France [8]. In 1965, the fungus was found in Berlin, Germany, on chopped maple wood after a city gardener, who had hackled a bigger batch of wood from sycamore, complained of respiratory irritations, vomiting and diarrhoea [9]. It was found that the in Germany previously not detected fungus must have grown under saprophytic conditions on the before stored maple trunks in a warm and humid cellar. Especially since the beginning of the twenty-first century, SBD has spread in several European countries: first reports 2004 near Vienna, Austria, 2005 in Baden-Wuerttemberg, Germany, and Prague, Czech Republic, 2011 in the Pays de la Loire region, France, and 2013 in the province of Noord-Holland, Netherlands [8]. In Italy, C. corticale was observed in 2013 in the northern Apennines near Montovolo [10]. In 2014, SBD was reported in Canton Geneva, Switzerland [11], and in Bulgaria in a park area near Sofia [12]. In 2018, the fungus was found in Belgium near Liège, afterwards in Antwerp, Charleroi and other localities [8].

Until now, the scientific literature about MBD as well as SBD and C. corticale is rather sparse. There are indications that outbreaks of SBD will become more frequent in the future due to climate change. In the last years, severe drought and heat periods, as well as prolonged flooding, resulted in higher outbreaks of the disease [12–14]. Therefore, it can be assumed that the resulting MBD, a hypersensitivity pneumonitis (HP) [15], will occur more frequently in the future. The risk group includes not only persons who have intensive occupational contact to infested maples or wood but also immunocompromised people [16]. This review takes up this topic providing recent research as well as insights of the past on SBD of maples, the fungus C. corticale and MBD.

### Sooty bark disease of maples caused by Cryptostroma corticale

**Fig. 1:**
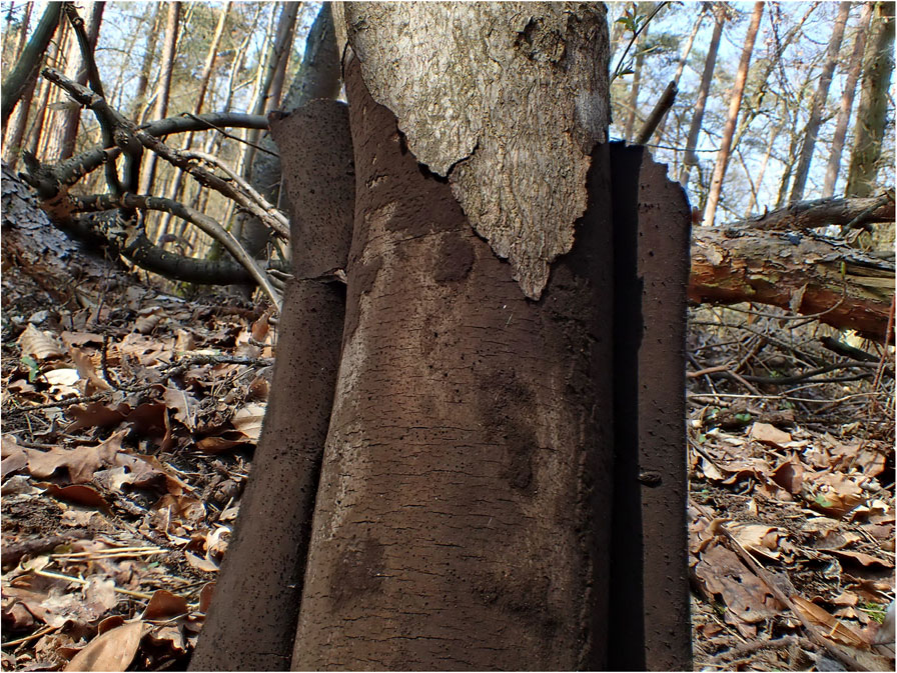
Trunk of an infested maple with sooty bark disease with characteristic bark peeling. Photo courtesy of Dr. Wolfgang Prüfert (German Mycological Society, DGfM e.V.)

**Fig. 2:**
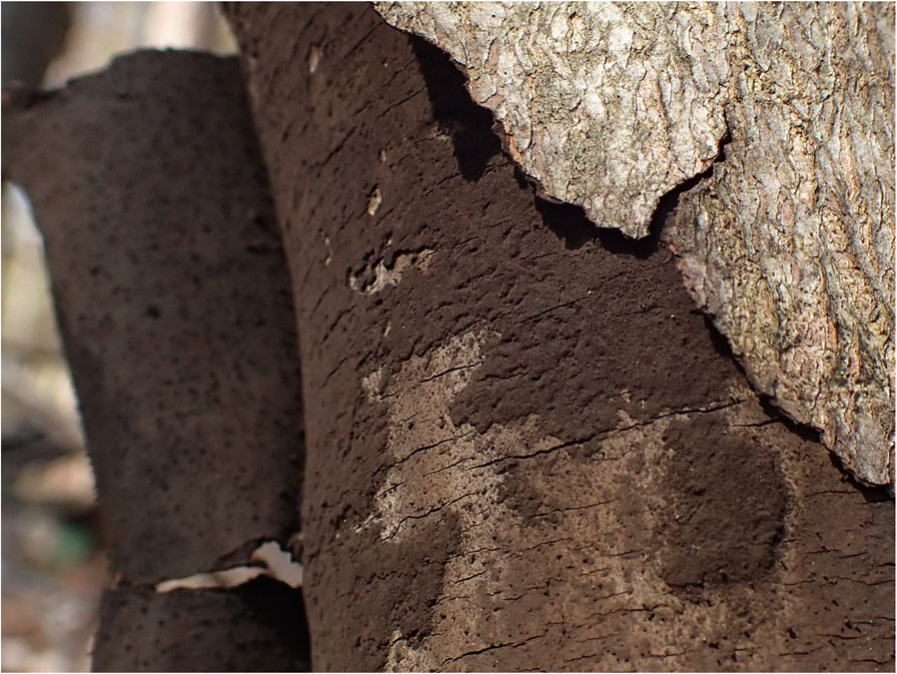
Planar soot-like coloured spore layer of Cryptostroma corticale on a maple trunk under the peeled bark. Photo courtesy of Dr. Wolfgang Prüfert (German Mycological Society, DGfM e.V.)

Sycamore (*Acer pseudoplatanus*) originated in mountainous areas of central Europe is a widespread tree in temperate zones of Europe and North America and belongs in central Europe to the most common trees in urban areas [13, 17]. The fungus C. corticale was first described by Ellis & Everhart in 1889 as a North American species and named Coniosporium corticale [18]. In 1951, Gregory & Waller changed the genus name of the fungus from Coniosprium to Cryptostroma because of some unique characteristics of the stroma and conidiophores [4]. The authors described SBD as a lethal disease of sycamore caused by C. corticale, a new in England introduced aggressive parasitical fungus. The disease is characterised by peeling of the outer layer of the bark and brownish-black spores under the peeled off bark. C. corticale appertains to the morphological fungus class hyphomycetes, often referred to as fungi imperfecti, anamorphic fungi or mould [19]. Based on their molecular data, Koukol et al. [13] have allocated C. corticale to the large genus Biscogniauxia within the Hypoxyloidae clade of Xylariaceae. A thesis of 1978 by S. D. Abbey at the Loughborough University of Technology [20], published in excerpts in the same year [21], described that C. corticale affects the maple through small openings or wounds. High summer temperatures facilitate the spreading of the fungus and the outbreak of SBD, while in years with normal temperatures and rainfall in summer, the disease process is without external visible symptoms [22]. The fungus mycelium grows from the portal of entry through the wood into the heartwood and spread lengthways without visible effects on the tree. This will be accelerated by drought stress [23]. At hot summers, the radial growth of the mycelium in the trunk increases [24]. When the mycelium attains the bark of the tree, the bark dies back and the spore layers are formed. The spores, first spreading in the trees’ xylem and later in the phloem, are produced in cavities of the fungal tissue with roof and floor stroma connected by stromatic columns leading to the death of the infested branches or the whole trunk [4]. The up to one centimetre thick spore layer, which coats the trunk and contains up to 170 million spores per cm^2^, can be spread by wind or washed off by rain [25]. Figure 1 show the infested trunk of a maple and Fig. 2 the planar soot-like coloured spore layer of C. corticale. Healthy trees can be asymptomatically affected by C. corticale as an endophyte while impairment of the tree, e.g., by drought may lead to a higher risk to contract on SBD [4, 26]. It is to assume that latent infections by C. corticale are widespread, also in urban plantations of maples [14].

It was observed, that outbreaks of SBD followed years with high summer temperatures [24, 27]. This was confirmed by growth experiments on C. corticale [23]. The study found in young sycamores that C. corticale grew better at 25 °C than at 15 °C and better in trees exposed to water stress. The thermophilic C. corticale profits of lack of water and heat stress in long-lasting summers [25]. Further stressors for the maples such as flooding, high nitrogen oxide concentrations and unfavourable on-site conditions also favour the spread of SBD [12–14]. It was found that urban conditions with climate extremes, higher temperatures and air pollution at traffic roads or paths lead to more SBD infection of sycamores [14].

In a risk analysis of C. corticale in Germany in 2018, the German Federal Research Centre for Cultivated Plants (Julius Kühn-Institut) declared that the fungus is meanwhile widespread and that the drought of 2018 in Germany will benefit the prevalence of SBD [28]. It is to point out that this year of drought in Germany was followed by the likewise dry year 2019. To date (effective September 2020), in Germany, the year 2020 appears also by drought [29]. In recent years, German public authorities informed increasingly about the spreading SBD [16, 30–32]. Also, the German medical journal Aerzteblatt indicated in 2019 that the tree disease SBD is spreading and that it is yet hardly investigated [33].

### Maple bark disease – a hypersensitivity pneumonitis

MBD was first named 1962 in a case report by Emanuel, Lawton and Wenzel [6]. The authors described MBD as a pneumonitis caused by C. corticale with classic symptoms of a farmer’s lung, but without its typical occupational exposure. In northern Wisconsin, USA, a 20-year-old male patient complained of dyspnea for 2 months, yellow sputum daily, frequent night sweats and a progressive loss of weight. The X-ray examination showed characteristics of interstitial pneumonitis. The histological diagnosis of the lung biopsy was an interstitial and focal granulomatous pneumonitis. No therapy was administered due to a fast improvement during the ten-day post-biopsy observation. The patient worked in a paper mill and sawed among others maple logs into short sections. Some maple logs were mouldy showing fine black dust under the outer bark. The black dust was identified as fungal spores of C. corticale identical to fungus cultured from the patients’ tissue obtained by the lung biopsy. In a survey of 37 men worked in that paper mill, five men had clinical symptoms of MBD, among them, four men had histological granuloma formation in the lung, but overall 16 workers were positive in serological tests showing sensitization to the spores of C. corticale [34]. In 1966, the same authors presented five cases of MBD [15]. They summarised that the unusual disease affects sycamore trees potentially leading to the death of the tree while at humans possibly leading to a totally disabling pulmonary disease by a hypersensitivity reaction due to spores of C.corticale.

In an immunological study on MBD published 1968, the immunological response of guinea pigs to spores of C. corticale was investigated [35]. It was found that the intradermal injection of spores and the inhaling of spores led both to circulating antibodies and skin reactivity after 24 h. The microscopic lung examination showed no granuloma formation at animals that received spores only intradermally. Five of 24 animals exposed to an airborne suspension of spores did so and, thus, showed symptoms of granulomatous disease. The study described that first symptoms and reaction on spore treatment appeared five or more hours after exposure with the hint that this is a long time for a hypersensitivity reaction. The authors ascribed this to the complex molecular structure of the antigen-antibody complex. Also, this study showed the often asymptomatic process after exposure to spores of C. corticale. It is to note that specific circulating antibodies to antigens inducing HPs like MBD indicate a sensitization, but not the disease, and are more useful as markers of exposure [36].

HP, also known as extrinsic allergic alveolitis (EAA), is characterised by the immunological respectively allergic reaction on repeated inhalation of organic antigens reaching the alveoli and provoking interstitial inflammation [37]. It is mostly a delayed immunoglobulin G (IgG) mediated reaction and not IgE mediated like in classical asthma [38]. Contemplable antigens are spores of fungi or bacterial, protozoal and animal proteins as well as low-molecular-weight molecules and metals [36, 39, 40]. Many diseases belong to HP, e.g., farmer’s lung and bird fancier’s lung respectively pigeon breeder’s disease, to name the most common ones, but also bagassosis, mushroom worker’s lung, malt worker’s lung and maple bark disease [41, 42]. The pathogenetic mechanisms of this often occupational lung disease are still not clear, but the sensitization process seems to play a decisive role [43]. Most antigen-exposed humans only develop a sensitisation without clinical significance and with a small local lymphocyte increase. However, antigen exposure can sometimes lead to an exacerbated immune reaction with inflammatory processes in the lung. Among others, type 1 helper cells and proinflammatory cytokines, e.g., tumour necrosis factor alpha (TNF-α), interleukin-12 (IL-12) and interferon gamma (INF-γ) will be released and persistent antigen exposure, as well as antibody production, can lead to critical alterations of the lung. The genetic predisposition can play a role in this overreaction of the immune system [36, 40, 44]. Until now, the processes that lead to disease progression and might culminating in lung fibrosis are not totally clarified [45].

Traditionally, HP was classified in acute, subacute and chronic forms based on duration and time course of the antigen exposure. Usually, the acute HP shows influenza-like symptoms a few hours after contact with a vast amount of antigen and a decrease in symptoms over hours or a few days without exposure. The occurrence of symptoms at re-exposure is frequent. The symptoms resemble acute respiratory infections. Sometimes, acute HP shows only mild or no respiratory symptoms or is associated with wheezing or bronchial hyperresponsiveness [36]. Repeated low-level inhalation of antigens over a long period may lead to a subacute HP with insidious symptoms of dyspnoea, cough and fatigue prolonging weeks or few months. The in general progressive persistent subacute HP can sometimes manifest oneself with acute episodes similar to a nonspecific febrile disorder with not until later nascent respiratory symptoms [36]. In acute and subacute HPs, chances of recovery are good by removing the triggering antigen and short-time treatment with corticosteroids [46–48]. Chronic HP can manifest when an acute or subacute HP is not recognised or remains untreated, and is characterised by progressive dyspnoea, cough, fatigue, malaise and loss of weight [36]. The presence of lung fibrosis on radiographic examination is the distinction to the subacute form. The development of severe irreversible physiologic impairments is common. Treatment of chronic fibrotic HP, in particular, is not consistent until now and often dependents on the opinions of experts and observations. A therapeutic approach at chronic HP might be consist of the administration of immunosuppressive drugs (e.g. corticosteroids) or antiproliferative drugs (e.g. azathioprine, mycophenolate mofetil or mycophenolic acid). In case of proliferation of the disease, antifibrotic treatment is a potential therapy [48, 49]. The progress of lung fibrosis is often associated with poor prognosis [46, 50]. Chronic HP can lead to a total loss of lung function culminating in death [40].

In recent years, the type of classification into three phenotypes was getting controversial and is possibly outdated in particular concerning the subacute type [51]. Lacasse and coauthors suggested the classification of HP into two clusters, because of overlapping symptoms of the subacute HP with symptoms of acute respectively chronic HP [52]. They reported based on data analysis of a large prospective multicentre cohort study HP patients with recurrent systemic symptoms like chills and body aches and normal chest radiography (cluster 1) and HP patients with hypoxemia, restrictive spirometry patterns and lung fibrosis on high-resolution computer tomography (HRCT) (cluster 2). A more recent classification of HP from 2017 by Vasakova et al. based on clinical-radiologic-pathologic correlations divides HP into the acute/inflammatory type (duration < 6 months, radiologic and histopathologic patterns, usually reversible) and the chronic/fibrotic type (prolonging or repetitive > 6 months, fibrotic changes in the lung) [51]. The authors pointed to the frequent misdiagnosis of HP and emphasised the need for more clinical studies aimed at a more solid diagnosis and better management of HP. A recently presented guideline for the diagnosis of HP classifies the disease into nonfibrotic and fibrotic phenotypes [53]. According to the authors, this guideline represents the first clinical practice guideline for clinicians to recognise nonfibrotic and fibrotic HP in adult patients with newly diagnosed interstitial lung disease (ILD).

The epidemiology of HP cannot be clearly specified due to, among others, a small number of studies, inconsistent definition of the disease and diagnostic methods. The variability of the prevalence is globally substantial, partly because of different geographical conditions, diverse antigen expositions and general risk factors of the potential affected. It is to assume that cases of HP might be not detected or misdiagnosed [36, 54, 55]. Although the data situation is poor, HP is considered a rare disease. For example, in the UK between 1991 and 2003 about 0.9 cases per 100,000 person-years could be detected [56, 57]. Other studies reported up to two cases per 100,000 persons [58, 59]. However, HP is a disease with a very high occupational amount [38].

The diagnosis of HP is intricate since lacking unique characteristics to the distinction compared to other ILDs [36]. Because of undiagnosed, misdiagnosed or delayed diagnosed cases of HP, the differentiation to diseases with similar symptoms including COPD, allergic asthma, flu or flu-like infections and interstitial pneumonia should be stand in focus. When applied successively, diagnostic methods including anamnesis, physical examination, serology, chest radiography or HRCT, lung biopsy, bronchoalveolar lavage (BAL) and specific inhalation challenges with qualifying antigens can lead to a possible diagnosis of HP [36, 38, 48, 53, 60–63]. For correct diagnosis and successful therapy, multidisciplinary expertise including pulmonologists, radiologists, pathologists and occupational physicians, to name a few, is helpful [53, 55, 64].

HP, and thus also MBD, is a recognised occupational disease in Germany, Austria and Switzerland, for example, and is notifiable in Germany [38]. Based on the key features of HP and the nomenclature for allergic diseases of the European Academy of Allergy and Clinical Immunology (EAACI), Quirce et al. proposed in an EAACI position paper the following summarising consensus definition for occupational HP (OHP): “OHP is an immunologic lung disease with variable clinical presentation and outcome resulting from lymphocytic and frequently granulomatous inflammation of the peripheral airways, alveoli and surrounding interstitial tissue which develops as the result of a non-IgE-mediated allergic reaction to a variety of organic or low molecular weight agents that are present in the work environment” [62].

### Risk assessment

Persons at risk are mainly people having intensive occupational contact with spores of C. corticale, e.g., woodman, foresters, sawyers or paper mill workers, but possibly also people with pre-existing lung disease or underlying allergic disease. To date, it is to assume that healthy walkers or mushroom pickers in forests or parks are not or only slightly vulnerable to getting MBD [16, 19, 31]. A case report on HP reported the contact of an affected orchid grower to C. corticale found in bark chips used as potting compost for orchids [65]. Probably, mouldy bark chips of maples in the compost were the source of the fungus. As the host of C. corticale, *A. pseudoplatanus*, is a common tree in parks and urban plantations, the contact to spores of C. corticale is not unlikely here for the public. In forests, where the tree is also common, as well as in paper mills and sawmills, there working persons are more affected.

By the German Mycological Society, in Germany, no cases of MBD are known yet (as from 27 March 2019), albeit this is to expect in the future due to evermore dry summers [19]. Therefore, it can be assumed that MBD will occur more frequently in the future not only in Germany but also in other countries with maple populations. Prospective, the medical relevance of MBD will increase.

In Central Europe, the survival of maples seems not to be endangered cause of the resistance of the trees [25]. However, from an economic point of view, the fungus C. corticale cause an almost total debasement of the maple wood even at an early stage of infestation [66].

### Recommendations for action and assessment for occupational safety

Generally, the most preventive measure as protection against HP is the avoidance or reduction of corresponding antigen exposure [62]. For patients with MBD, most important is removing of the causal host of C. corticale, e.g., infested wood of maples, respectively avoiding further exposure minimising the risk of progression of the disease. Usually, patients with acute HP recover after antigen avoidance [36]. Crucial, a profound and timely diagnosis of HP and the identification of the causal antigen should be preceded that lead to an improvement of the therapy [51].

A study from Prague, Czech Republic, provided indications that for prevention of SBD of maples, and, as a consequence, of MBD of humans, removing of infested trees may slow down the spreading of the disease [14].. Indeed, less A. pseodoplatanus trees with obvious SBD were found in parks than expected after the park management of Prague removed diseased trees a few years before. On the other side, the same study detected a high grade of latent C. corticale infections using early detection methods (fungus cultivation from wood tissue and PCR with species-specific primers). As latent infections of urban maples are present, a preventive measure should be the avoidance of stress to the trees by the warranty of water supply, which reduces the vulnerability to SBD [25]. Additionally, preventive strategies are the wide cordoning of infested areas to protect the public and the immediate removal of diseased trees. For occupational safety to protect the forestry worker from fungal spores, at all works on infested trees, the personal protective equipment (goggles, filtering facepiece, overall, protective gloves and boots) is to wear. Felling of trees should be performed by machine and at damp weather [25]. Since the sporulation of C. corticale is lowest in winter, the felling in the cold season is recommended [12]. The infested wood should be buried locally or transported covered in containers with subsequent burning in a, e.g., waste incinerator. However, the infested wood is not suitable as firewood and must not be chipped to avoid spreading of spores [25, 67]. For this purpose, the German Social Insurance for Agriculture, Forestry and Horticulture (SVLFG) has provided an exemplary directive [68].

## Conclusion

SBD of maples, which originates from North America, is meanwhile also widespread in Europe, especially in England and Central Europe. As the disease provoking fungus C. corticale benefits from hot summers and host trees weakened by drought, the ongoing climate change will expedite the further spreading. Consequently, the risk for humans to contract on MBD will increase. Since MBD like all HPs as a complex syndrome is often undiagnosed, misdiagnosed or delayed diagnosed, stricter diagnostic criteria and multidisciplinary networking are essential to improve diagnosis and therapy. If MBD is diagnosed in time, in most cases the removal of the causative fungus or the infested maple wood, respectively, lead to complete recovery. Hence, further research is required to develop diagnostic tools for the rapid and reliable identification of the disease trigger.

## Data Availability

Not applicable.

## Abbreviations

A: Acer
BAL: Bronchoalveolar lavage
C. corticale: Cryptostroma corticale
COPD: Chronic Obstructive Lung Disease
EAA: Extrinsic allergic alveolitis
EAACI: European Academy of Allergy and Clinical Immunology
HIV: Human immunodeficiency virus
HP: Hypersensitivity pneumonitis
HRCT: High-resolution computer tomography
Ig: Immunoglobulin
IL: Interleukin
ILD: Interstitial lung disease
INF-γ: Interferon gamma
MBD: Maple bark disease
OHP: Occupational hypersensitivity pneumonitis
PCR: Polymerase chain reaction
SBD: Sooty bark disease
SVLFG: Sozialversicherung für Landwirtschaft, Forsten und Gartenbau
TNF-α: Tumour necrosis factor alpha
